# Synergistic Effects of Ultrasound and pH-Shifting on the Solubility and Emulsification Properties of Peanut Protein

**DOI:** 10.3390/foods14050853

**Published:** 2025-03-02

**Authors:** Zhuoran Jiao, Zhiqiang Feng, Siqi Zhao, Yuwei Wang, Miao Feng, Qian Chen, Baohua Kong, Haotian Liu

**Affiliations:** College of Food Science, Northeast Agricultural University, Harbin 150030, China; jzr0100@163.com (Z.J.); ffcyfzq@163.com (Z.F.); zhaosiqi0121@163.com (S.Z.); 13682101156@163.com (Y.W.); 17866928220@163.com (M.F.); chenqianego7@126.com (Q.C.); kongbh63@hotmail.com (B.K.)

**Keywords:** peanut protein, solubility, emulsification, pH-shifting, high-intensity ultrasound

## Abstract

Peanut protein is a byproduct of peanut oil extraction with limited applications within the food sector due to its low solubility and emulsifying properties. This study investigated the influences and mechanisms of high-intensity ultrasound (HIU, 200~600 W) and pH-shifting (pH 12), either individually or jointly, on the structure, solubility, and emulsifying properties of PP. Results indicated that the solubility of PP significantly increased after the combined treatment, particularly when the HIU power was 300 W (*p* < 0.05). Accordingly, emulsions prepared from it exhibited highest storage stability. Structural analysis indicated that the increased PP solubility (9.95% to 54.37%, *p* < 0.05) is mainly attributed to the structural changes that occur during protein unfolding, resulting in the uncovering of hydrophobic groups (7181.43 to 14,083.00, *p* < 0.05) and the reduction of α-helices (24.43% to 18.17%, *p* < 0.05). Moreover, confocal laser scanning microscopy of the emulsions revealed that the combination-treated PP resulted in smaller protein particle sizes (50.09 μm to 15.68 μm, *p* < 0.05), tighter adsorption on the oil–water interface, and a denser and more stable interfacial film compared to the native and the individual treatment, thereby enhancing the stability of the system. A rheological analysis confirmed that the combined treatment improved the interfacial properties of the protein, which was advantageous for emulsion stability. In conclusion, HIU combined with pH_12_-shifting can appreciably improve the solubility and emulsifying properties of PP to broaden its application prospects.

## 1. Introduction

Peanuts (*Arachis hypogaea* L.) are rich in oil and protein, and as a widely cultivated crop worldwide, they are the third primary source of plant protein in the human diet [1]. Peanut proteins, as a byproduct of the peanut oil extraction process, constitute approximately 47% to 55% of defatted peanut meal, are rich in essential amino acids, low in anti-nutritional factors, with an effective utilization rate of more than 90%, and have a nutritional value that is roughly equivalent to that of animal source proteins [2,3]. However, PP’s high molecular weight and limited number of ionizable groups leads to its low solubility in aqueous media and directly affects its emulsifying capabilities, thereby significantly restricting its practical applications in food production [4].

The limited functionality of PP can be ascribed to its highly organized quaternary structure, intermolecular hydrophobic interactions, and disulfide cross-links (S-S), hindering its water dissolution and effective localization at oil–water interfaces of emulsions. Various physical and chemical methods for protein modification have been developed to address this, including high pressure, microwave treatment, glycosylation, phosphorylation, and enzymatic hydrolysis [5,6]. However, single-method treatments have limited efficacy, causing a shift toward combined methodologies for optimizing protein structure and enhancing its functional properties. For example, M. Wang et al. [7] modified peanut protein isolate by enzymatic treatment combined with glycosylation, which altered the protein’s spatial structure and enhanced its flexibility, thereby improving its emulsifying properties. Chen et al. [8] modified PPI through extrusion cooking pretreatment and papain-induced proteolysis, resulting in a notable enhancement in the hydrolysis extent and protein solubility of the hydrolysate, along with a marked improvement in emulsification. However, these methods are frequently cumbersome to operate and possess various degrees of limitations. For instance, the degree of the glycosylation reaction is not easy to control. It also requires strict high-temperature conditions, which promote the production of carcinogenic byproducts and lead to nutrient degradation [9]. Enzymatic hydrolysis has the disadvantages of high enzyme cost, long hydrolysis time, and low yield [10]. Therefore, better methods are needed to improve the solubility and emulsification of PP.

Ultrasonic waves carry mechanical energy through the local vibration of particles at frequencies exceeding 20 kHz. They propagate through the material, especially liquid media, and reflect off walls and internal reflectors, such as volumetric voids and inclusions [11]. High-intensity ultrasound (HIU) is a nonthermal, physically-based treatment technology known for its simplicity, green nature, and lack of pollution. It has gained widespread adoption for modifying food proteins. Acoustic cavitation, induced by HIU, is a periodic phenomenon involving rapid bubble expansion and violent collapse, which generates substantial mechanical forces, including shear forces, shock waves, and microturbulence. These mechanical forces lead to the disintegration of protein particles and aggregates, exposing the hydrophobic and sulfhydryl groups (-SH), thus improving the functional attributes of the proteins, including their aqueous solubility, emulsification, and foaming properties [12,13]. However, HIU can only impact large-scale aggregation of rigid proteins by altering intermolecular forces, exhibiting minimal effect on intramolecular forces and, consequently, failing to modify the protein’s inherent rigid structure and, thus, has limited effect on improving protein function [14].

pH-shifting, recognized as a straightforward, mild, and effective chemical modification technique, has been extensively documented to substantially enhance the functional characteristics of proteins. By altering the pH of the medium, the protein is exposed to extreme acid-base environments, leading to its unfolding. Subsequently, the pH is readjusted to neutral, allowing the protein to refold. This process may lead to the formation of a flexible structure termed a “molten globule” [15]. Yang et al. [16] explored the impact of pH-shifting on egg yolk protein (EYP) and observed that alkaline pH-shifting increased the number of hydrophobic groups exposed in EYP, modifying its tertiary structure. Furthermore, the pH-shifting treatment decreased the size of EYP particles in the solution and improved the protein emulsification properties. Liu et al. [17] demonstrated that pH-shifting causes proteins to unfold, exhibit greater flexibility, expose more hydrophobic groups and amino acids, and thus increase protein complexation with curcumin.

Although HIU and pH-shifting have shown potential in protein modification, their combined effects on PP remain underexplored. Specifically, the synergistic mechanisms of HIU and pH-shifting in modifying the structural and functional properties of PP are not well understood. This knowledge gap limits the development of efficient and scalable modification strategies for industrial applications. Against this background, we propose the hypothesis that the application of HIU during pH-induced protein unfolding disrupts the flexible molecular chains through the cavitation effect. Specifically, after pH-shifting treatment, the protein structure undergoes unfolding, resulting in a more flexible conformation. When HIU is applied, the mechanical forces and cavitation effects act more effectively on disrupting the intermolecular linkages between the protein chains. The subsequent readjustment of the pH to neutral facilitates the flexible refolding of protein units, resulting in the formation of protein particles with adaptable conformations. This process is anticipated to markedly enhance the solubility of PP, thereby improving its functionality within emulsion systems.

Building on the aforementioned background, this study investigates the influences of HIU and pH-shifting, either separately or jointly, on the solubility and emulsification properties of PP. Furthermore, investigations into structural alterations were conducted to reveal the mechanisms underpinning the enhancements in solubility and emulsifying properties. The aim is focused on substantiating our hypothesis and broadening the application of PP in the field of foodstuffs through these theoretical insights.

## 2. Materials and Methods

### 2.1. Materials

The PP powder (protein ~55%) was bought from Jiangsu Yuanshengtong Bioengineering Co., Ltd., Nanjing, China and stored at 4 °C. Peanut oil was purchased from Shandong Luhua Group Co., Ltd., Yantai, China. All other chemicals and reagents used in this study were of analytical grade.

### 2.2. Ultrasonic Treatment and pH-Shifting

The PP powder (50 mg/mL) was dispersed in deionized water at 4 °C with constant agitation for an extended period to ensure thorough hydration and uniformity. Subsequently, according to the result of the preliminary experiments, the PP suspension underwent pH_12_-shifting, as detailed by Zhang et al. [14]. In brief, the pH of the PP suspension was raised to 12 using 2 M NaOH while maintaining continuous stirring, and the suspension was maintained at this pH for 2 h to ensure reaction homogeneity. During pH adjustment, the PP suspensions were HIU-treated using an ultrasonic cell pulverizer (Scientz-IID, Ningbo Scientz Biotechnology Co., Ltd., Ningbo, China) with a 6 mm diameter probe at 20 kHz and various power levels (200, 300, 400, 500, and 600 W) for 5 min (on-time 2 s and off-time 3 s pulse duration). Throughout the HIU process, the sample was consistently maintained in a chilled water bath to ensure its temperature remained below 20 °C, with real-time monitoring conducted using an integrated temperature probe. Following HIU treatment, the PP solution was stirred for 1 h at 25 °C and then pH-adjusted to 7.0 using 1 M HCl. PU_2_, PU_3_, PU_4_, PU_5_, and PU_6_ represent samples subjected to pH-shifting and HIU at various powers of 200, 300, 400, 500, and 600 W, respectively. To assess the joint impact of the two treatments, we prepared a series of samples treated by pH-shifting and HIU (at various powers) alone, under the same conditions described above, respectively labeled as P, U_2_, U_3_, U_4_, U_5_, and U_6_. “C” represents the control group of the untreated native protein. All samples were kept at 4 °C for subsequent analysis.

### 2.3. Solubility Determination

Solubility was assessed according to the modified method of Ma et al. [18]. After diluting the sample (50 mg/mL) to 10 mg/mL using deionized water, it was centrifuged at 10,000× *g* for 15 min. The Biuret method was used to measure the protein concentration in the supernatant. Protein solubility was determined using the following formula:

(1) $$Solubility\;(\%)=\frac{Supernatant\;protein\;concentration\;(mg/mL)}{Total\;protein\;concentration\;(mg/mL)}\times 100\%$$

### 2.4. Particle Size and Zeta Potential Determination

A Zetasizer Nano-ZS 90 (Malvern Instruments, Worcestershire, UK) was used to assess the particle size, polydispersity index (PDI), and zeta potential of the samples. Before measurement, the samples (50 mg/mL) were diluted to 0.1 mg/mL using deionized water and centrifuged at 10,000× *g* for 15 min to minimize multiple scattering effects [11]. All experiments were performed at 25 °C.

### 2.5. Microscopic Morphological Characterization

The micromorphology and distribution of PP under various treatments were observed using confocal laser scanning microscopy (CLSM; Leica TSC SP8, Leica Microsystems, Heidelberg, Germany). The procedure involved diluting the samples (50 mg/mL) to 10 mg/mL with deionized water, incorporating 20 μL of a protein staining solution (1.0 mg/mL Nile blue solution) into 1.0 mL of a protein suspension, vortexing for 2 min, and then allowing the reaction to proceed in the dark for 30 min. Afterward, 1.5 mm thick microscope slides were coated with 10 μL of the stained samples and covered with coverslips. The distribution of PP was then examined under a 40× objective lens using a 633 nm He-Ne laser as the light source [19].

### 2.6. Sodium Dodecyl Sulfate-Polyacrylamide Gel Electrophoresis (SDS-PAGE) Analysis

Referring to the approach of Ji et al. [20] with modifications, sodium dodecyl sulfate-polyacrylamide gel electrophoresis (SDS-PAGE) was conducted on PP samples subjected to various treatments. An equal volume of loading buffer (0.05 M Tris-HCl, 10% glycerol, 2% SDS, 0.02% bromophenol blue; pH 6.8) with or without 12.5 mM β-mercaptoethanol was added to the protein samples (2.0 mg/mL), followed by boiling for 3 min. SDS-PAGE was performed using 10% separating and 5% concentrating gels at a constant voltage of 120 V. The gels were stained with Coomassie brilliant blue R-250 for 10 min and decolorized using a decolorizing solution (5% methanol and 7.5% acetic acid).

### 2.7. Structural Characterization of PP

#### 2.7.1. Fourier Transform Infrared (FTIR)

Fourier transform infrared (FTIR) spectroscopy was used to determine the secondary structure of the protein. The protein sample suspension was freeze-dried for 12 h to yield a lyophilized powder. The FTIR spectra of the powder samples were recorded using a Nicolet iS50 (Thermo Fisher Scientific, Shanghai, China) over a wavelength range of 4000–400 cm^−1^ [21]. Spectra analysis was performed using OMNIC (Analytical Software, Waltham, MA, USA) and PeakFit software (Analytical Software, San Jose, CA, USA) to obtain the percentage of secondary structure for the samples after different treatments.

#### 2.7.2. Intrinsic Fluorescence Spectroscopy

An RF6000-fluorescence spectrophotometer (Hitachi Co., Tokyo, Japan) was used to measure the fluorescence intensity of the samples (0.1 mg/mL, diluted from 50 mg/mL with deionized water) after different treatments, with an excitation wavelength of 280 nm and emission wavelength between 250 and 400 nm [22]. The slit widths for both excitation and emission were set at 10 nm, and the scanning speed was set to 6000 nm/min.

#### 2.7.3. Surface Hydrophobicity (H_0_)

For evaluating the surface hydrophobicity (H_0_), an established protocol [11] was used with minor modifications. Specifically, the samples (50 mg/mL) were diluted with deionized water to achieve concentrations between 0.2 and 1.0 mg/mL. Into 4 mL of each diluted sample, 20 μL of ANS solution (pH 7.8, 8 mM) was introduced, followed by a 30 min reaction period in the dark. The fluorescence intensity was recorded at an excitation wavelength of 380 nm and an emission wavelength of 480 nm; the slit width was 5 nm and the scanning speed was 6000 nm/min. H_0_ was quantified by determining the initial slope of the fluorescence intensity versus protein concentration (mg/mL) plot, as determined by linear regression analysis.

#### 2.7.4. Sulfhydryl Groups (-SH) and Disulfide Bonds (S-S)

The -SH and S-S contents in the samples were assessed using a method adapted from Yang et al. [23]. To determine the free -SH content, 1 mL of protein sample (1 mg/mL) was mixed with 4 mL of Buffer B (0.086 M Tris, 0.09 M Gly, and 4 mM Na_2_EDTA). To assess the total -SH content, 1 mL of protein sample (1 mg/mL) was mixed with 4 mL of Buffer B+ (Buffer B supplemented with 0.5% SDS and 6 M urea). Subsequently, 50 μL of Ellman’s reagent (4 mg/mL DTNB dissolved in Buffer B) was introduced into each sample, thoroughly mixed, and allowed to react in the dark for 30 min. After centrifugation of the samples at 10,000× *g* for 10 min, the absorbance of the supernatant was measured at 412 nm. The -SH content of the sample was obtained from Equation (2):

(2) $$-SH\;(µmol/g)=\frac{73.53\times A\times D}{C}$$

where *A* represents the absorbance at 412 nm, *D* denotes the dilution factor, and *C* indicates the protein concentration (mg/mL).

The S-S content of the samples was obtained from Equation (3):

(3) $$\mathrm{S-S}\;(µmol/g)=\frac{Total-SH-Free-SH}{2}$$

### 2.8. Emulsifying Properties Characterization

#### 2.8.1. Preparation of PP Emulsions

Emulsions were prepared following a modified version of the method reported by Zhang et al. [14]. Ten milliliters of peanut oil were mixed with 90 mL of PP suspensions that had undergone various treatments (C, U_3_, P, PU_2_, PU_3_, and PU_4_). The mixture was homogenized at 12,000 rpm for 2 min using a T18 basic high-speed homogenizer (IKA Works GmbH & Co., Staufen, Germany) to form a crude emulsion and then passed twice through a high-pressure homogenizer (SPCH-10; Shanghai Donghua High Pressure Homogenizer Factory, Shanghai, China) at 70 MPa. The resultant emulsions were stored at 4 °C for 7 days to evaluate their storage stability, and the appearance of the emulsions was documented during this time.

#### 2.8.2. Particle Size and Zeta Potential of PP Emulsions

The particle size and droplet size distribution of both freshly prepared and 7-day-stored emulsions were assessed using a SYNC laser particle sizer (Microtrac Inc., York, PA, USA). Zeta potential measurements of the emulsions were undertaken using a Zetasizer Nano-ZS 90 (Malvern Instruments). Prior to measurement, the samples were diluted 100-fold using deionized water.

#### 2.8.3. Characterization of the Microstructure of PP Emulsions

CLSM (Leica TSC SP8, Leica Microsystems, Heidelberg, Germany) was implemented to assess the micromorphology and distribution patterns of emulsions stabilized by PP with different treatments. To this end, 20 μL of Nile blue (0.1% *w*/*v* dissolved in water) and 25 μL of Nile red (0.1% *w*/*v* dissolved in alcohol) were blended into 1 mL of the emulsion and vortexed for 2 min, then allowed to react in the dark for 30 min. Next, 10 μL of the stained sample was applied to 1.5 mm thick microscope slides and covered with a coverslip. The micromorphological distribution of the emulsions was observed under a 40× objective lens using 488 and 633 nm excitation wavelengths as light sources [24,25].

#### 2.8.4. Rheological Properties of PP Emulsions

The rheological properties of the emulsions were measured using a modular rotational rheometer (HAAKE MARS60, Thermo Fisher Scientific, Shanghai, China). Apparent viscosity measurement was taken at shear rates from 0.01 to 100 s^−1^. Dynamic viscoelasticity was evaluated in the linear viscoelastic region from 0.1 to 10 Hz to record the storage modulus (G′) and loss modulus (G″) [26].

### 2.9. Statistical Analysis

All experiments were repeated three times. All data are expressed as mean ± standard deviation (SD). SPSS Statistics 27 software (Analytical Software, Armonk, NY, USA) was used for statistical analysis. Analysis of variance (ANOVA) was used to test the significance of the data. Tukey’s test was used for multiple comparisons with the significance level set at *p* < 0.05. All graphs were plotted using Origin 2023b software (Analytical Software, Northampton, MA, USA).

## 3. Results and Discussion

### 3.1. Solubility

**Figure 1:**
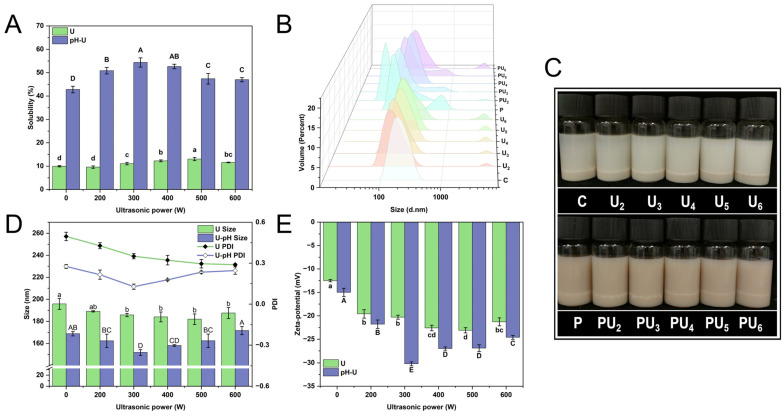
(**A**) solubility, (**B**) droplet distribution, (**C**) appearance, (**D**) mean particle size and polydispersity index (PDI), and (**E**) zeta potential of native (C) and treated PP with high-intensity ultrasound (HIU, 200–600 W) and pH-shifting (pH 12), individually (U_2_, U_3_, U_4_, U_5_, U_6_, and P) or in combination (PU_2_, PU_3_, PU_4_, PU_5_, and PU_6_). U and U-pH represent HIU treatment alone and the combined treatment of HIU and pH-shifting, respectively. Different upper (A–E) and lower (a–d) case letters denote marked differences in the solubility, size, and zeta potential of PP with HIU individual and pH-shifting and HIU combined treatment, respectively (*p* < 0.05).

Solubility represents a critical characteristic of proteins, with insufficient solubility potentially impacting other functional attributes, including emulsification, foamability, and gelation [27]. As illustrated in Figure 1A, the solubility of native PP is low (9.95%) because the protein’s highly ordered, rigid three-dimensional (3D) structure promotes aggregation into insoluble particles with a hydrophobic core, resulting in poor functional properties [28]. During the pH-shifting treatment, PP experiences partial unfolding and assumes a flexible molten globule conformation under highly alkaline conditions, leading to a marked increase in solubility (*p* < 0.05). The mechanical effect of acoustic cavitation during HIU treatment enhances the solubility of PP (Figure 1A), although this enhancement is less significant compared to pH-shifting (*p* < 0.05). Previous studies have extensively reported that the intense mechanical forces generated by HIU can decrease the size of protein particles and increase the specific surface area, thereby enhancing the hydration capacity [29,30]. However, for highly ordered globular proteins, despite the reduction in particle size, aggregation remains inevitable due to the formation of a hydrophobic core, which is the driving force limiting the solubility of PP post-HIU treatment. These results are consistent with the findings of our previous research [14].

The significantly higher solubility of PU_3_, reaching a maximum of 54.37% (*p* < 0.05) compared to other treatments can be attributed to the synergistic effects of pH-shifting and HIU, which collectively enhance protein solubility through multiple mechanisms (Figure 1A). Under alkaline conditions (pH 12), strong electrostatic repulsion between protein molecules weakens intermolecular interactions, causing the protein to swell and adopt more flexible secondary and tertiary structures [31]. This expanded conformational state exposes more hydrophilic groups to the aqueous environment, thereby enhancing solubility. Furthermore, HIU contributes to this process through transient cavitation effects, including robust mechanical shear forces and localized high temperatures and pressures. These forces disrupt the flexible polymer structure, preventing protein refolding and altering hydrogen bonds, hydrophobic interactions, electrostatic forces, and other non-covalent bonds. As a result, the protein undergoes conformational changes that increase its spatial expansion at the protein–water interface, as reported by Ma et al. [18], ultimately enhancing solubility. Specifically, in PU_3_, the combination of optimal HIU power (300 W) and pH-shifting creates a unique environment where the protein structure is sufficiently expanded without causing reaggregation. However, the solubility enhancement is HIU power-dependent (Figure 1A), with solubility decreasing when the power exceeds 300 W (*p* < 0.05). This may be attributed to protein reaggregation induced by the excessive HIU power, which counteracts the desired effects of pH-shifting and leads to a decrease in solubility [32]. The combined treatment (PU_3_) thus achieves an optimal balance between structural flexibility and mechanical disruption, maximizing solubility while avoiding overprocessing-induced reaggregation.

Figure 1C illustrates the visual appearance of PP suspensions subjected to various treatments. Native PP (C) suspensions exhibited significant sedimentation at the bottom of the bottle, indicating pronounced phase separation and thus demonstrating low solubility and poor stability. In contrast, the PP suspensions treated with HIU alone showed no noticeable changes in appearance, suggesting that individual HIU treatment had minimal effects. Upon applying the synergistic effect of pH_12_-shifting and HIU at various intensities, the suspension’s color intensified compared to native PP (C). This indicated a more homogeneous distribution of particles within the solution, consistent with the solubility results.

### 3.2. Particle Size and Zeta Potential

Particle size and zeta potential are essential for deciphering the intrinsic correlation between protein–protein interactions and their structural attributes. Figure 1B,D display the particle distribution, size, and PDI of PP following various treatment protocols. Among the various treatments, native PP has a larger particle size due to the presence of a higher number of insoluble aggregates. When HIU treatment was applied alone, the particle size decreased gradually, from 195.77 ± 4.90 to 181.93 ± 4.76 nm, as the ultrasonic power increased (*p* < 0.05). This is attributed to ultrasonic cavitation. Cavitation-induced shear stress and turbulence augment the frequency and intensity of PP collisions, potentially disrupting the intermolecular interactions essential for maintaining the structure of protein aggregates in aqueous solution [33,34]. Despite the particle-shearing stresses associated with acoustic cavitation, subsequent hydrophobic aggregation remains inevitable, resulting in limited particle size reduction. When treated by a combination of pH-shifting and HIU, the particle size of PP was markedly diminished and exhibited a more homogenous distribution compared to PP treated with either approach individually (Figure 1B,D). PU_3_ exhibited the most diminutive particle size and the most homogeneous distribution (*p* < 0.05), which was in concordance with the solubility outcomes (Figure 1A).

Research has demonstrated that the application of ultrasound in conjunction with acid treatment can markedly diminish the particle size of pine kernel protein, consequently improving its solubility [35]. Similarly, Yang et al. [36] demonstrated that their coprocessing method effectively reduced the particle size of perilla protein from 1218.30 to 71.88 nm, ascribing this reduction to the significant mechanical forces exerted by HIU (900 W for 20 min) and the electrostatic repulsion elicited by pH-shifting. Hence, the discernible decrease in particle size can be ascribed to the subsequent factors: firstly, in highly alkaline conditions, the protein expands and exposes reactive groups, which facilitates the formation of a flexible structure. Then, the vigorous physical forces exerted by HIU disrupt various non-covalent bonds, such as hydrogen bonds, electrostatic interactions, and hydrophobic forces, leading to the disintegration of the flexible structure into smaller protein particles [19]. Additionally, the pH of the alkaline environment deviates considerably from the protein’s isoelectric point, at which PP exhibits strong electrostatic repulsion. Concurrently, the HIU treatment enhances the collision frequency among unstable protein aggregates. These superposition effects promote the uniform distribution of protein particles in water [2,35,37].

Regardless of whether HIU is used alone or in combination, however, when the ultrasound power is too large, a significant increase in particle size is observed, particularly for PU_6_, which exhibits a prominent peak in the large particle size distribution. During HIU treatment, the decomposition of water generates highly reactive free radicals, which can cause protein oxidation to a certain extent, leading to intermolecular cross-linking and the formation of aggregates, potentially explaining the large particle distribution peak [38]. Accordingly, it is hypothesized that an excessively high HIU power might instigate uncontrollable cavitation, generate highly reactive radical species, provoke intense and excessive collisions of particles, and induce internal excessive heating, which could ultimately lead to the reaggregation of previously dissociated aggregates. The findings align with those reported by Yang et al. [39], which indicated that ultrasound treatment of quinoa protein at 800 W for 20 min led to an increase in particle size.

The zeta potential indicates the surface charge of proteins. Although electrostatic repulsion is influenced by multiple factors, a greater absolute value of the zeta potential typically leads to increased repulsion, thereby enhancing the stability of the protein system and reducing its propensity for aggregation [40]. As shown in Figure 1E, native PP displays a relatively low net negative charge (*p* < 0.05) attributed to the deprotonation of carboxyl groups. After treatment, the overall negative zeta potential value exhibited a significant increase (*p* < 0.05), regardless of whether PP was treated by HIU alone, pH-shifting alone, or a combination of these two treatments. HIU can induce physical shearing that disrupts protein structure, resulting in the exposure of polar residues [41]. In contrast, pH-shifting induces protein refolding, exposing more negative charges and intensifying electrostatic repulsion on PP surfaces. The resulting looser and more flexible molecular structure enhances the sensitivity of PP to subsequent HIU treatment, allowing the mechanical forces and cavitation effects to more effectively disrupt intermolecular interactions and further modify the protein structure [42]. Hence, as the polar groups on the surface ionize, the molecular surface accumulates charges, resulting in an increased net negative charge. Systems with a substantial net negative charge generally display diminished aggregation and augmented stability, attributable to the robust electrostatic repulsion force between protein particles, as reported by Hussain Badar et al. [43]. This is in agreement with the data presented in Figure 1A,B. Similarly, when the ultrasonic power was too large, the increased intensity and subsequent thermal effects led to the reaggregation of the dispersed system, resulting in the reburial of polar groups previously exposed on the protein’s molecular surface.

### 3.3. Confocal Laser Scanning Electron Microscopy (CLSM)

**Figure 2:**
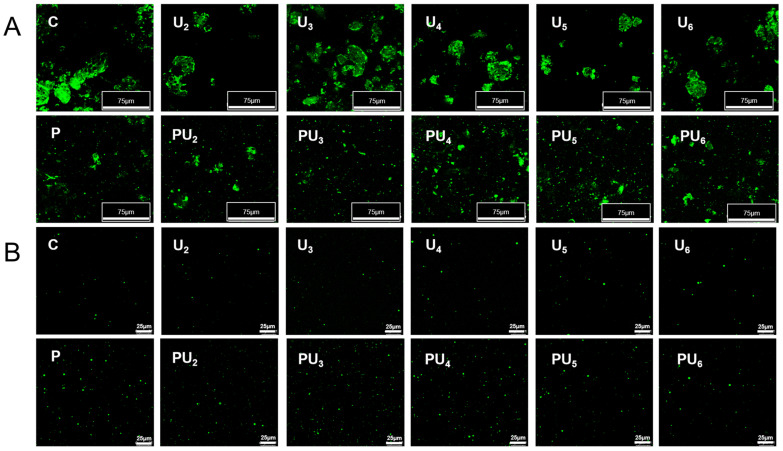
Confocal laser scanning microscopy (CLSM) micrographs of (**A**) the suspensions and (**B**) the supernatant of native (C) and treated PP with high-intensity ultrasound (HIU, 200–600 W) and pH-shifting (pH 12), individually (U_2_, U_3_, U_4_, U_5_, U_6_, and P) or in combination (PU_2_, PU_3_, PU_4_, PU_5_, and PU_6_).

The microstructure and distribution of PP were imaged using CLSM, and the results are illustrated in Figure 2A. Native PP possesses a dense, irregular protein structure, with large particles and a heterogeneous particle size distribution in the protein suspension. The structure contains numerous cavities, which may be remnants of lipid globules from the peanut cells [44]. Upon HIU treatment, the structure of the PP unfolds and becomes looser, with mechanical shear forces fragmenting the protein and causing a slight reduction in particle size. In comparison, pH-shifting has a more direct effect, as it leads to a marked reduction in protein particle size and results in a system that is more uniform and better dispersed. Subsequent HIU generates mechanical shear forces and turbulence, which further destroy the interactions, such as hydrogen bonding and hydrophobic forces, within the protein, leading to particle morphology changes in PP [45]. Consistent with the preceding solubility findings (Figure 1), PU_3_ exhibited smaller and more uniformly distributed particles compared to other treatments. However, when the HIU power in the combined treatment exceeded 400 W, PP exhibited reaggregation and increased particle size, aligning with the findings of Sun et al. [46], who proposed that extended ultrasonication (3.17 W/cm^3^, 50 min) might induce the reassembly and stacking of protein particles.

Figure 2B illustrates the changes in PP in the supernatant following various treatments. Consistent with the solubility results (Figure 1A), native PP has a low content of soluble protein and an uneven size distribution. The HIU treatment alone slightly increased the content of soluble protein. However, after the combination of pH-shifting and HIU treatment, a system with a higher content of soluble protein and uniform size dispersion was obtained. Similarly, Dabbour et al. [47] have reported that the synergistic application of pH-shifting and ultrasound treatment markedly augments the solubility of cottonseed protein. They attributed this to the exposure of polar groups following alkaline pH changes, leading to the formation of a molten globule structure, which increases protein molecular flexibility. Subsequent ultrasound introduces cavitation-induced mechanical effects, potentially altering the 3D structure of globular proteins, reducing their particle size, and ultimately improving interactions with water molecules. Moreover, the increase in local pressure and temperature during ultrasound treatment leads to further protein unfolding, promoting the exposure of more hydrophilic amino acids, thereby converting initially insoluble aggregates into more soluble forms [48].

### 3.4. Sodium Dodecyl Sulfate-Polyacrylamide Gel Electrophoresis (SDS-PAGE)

**Figure 3:**
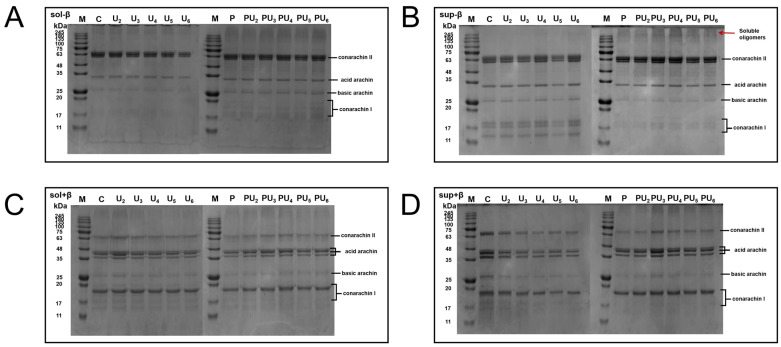
Sodium dodecyl sulfate-polyacrylamide gel electrophoresis (SDS-PAGE) pattern of (**A**,**C**) solutions and (**B**,**D**) supernatants for native (C) and treated PP with high-intensity ultrasound (HIU, 200–600 W) and pH-shifting (pH 12), alone (U_2_, U_3_, U_4_, U_5_, U_6_, and P) or in combination (PU_2_, PU_3_, PU_4_, PU_5_, and PU_6_) under (**A**,**B**) nonreducing and (**C**,**D**) reducing conditions. M indicates the marker.

The SDS-PAGE results of the PP solution and supernatant are shown in Figure 3. Approximately 90 wt% of PP are globular proteins, which can be categorized into three primary groups: arachin (66 wt% of the PP), conarachin I (13 wt%), and conarachin II (16 wt%) [49]. From Figure 3A, in the absence of β-mercaptoethanol, all samples displayed the typical electrophoretic band pattern of PP, with visible bands characteristic of conarachin II (67.0 kDa), acid arachin (35.5 kDa), basic arachin (21.6 kDa), and conarachin I (18.0, 17.0, and 15.5 kDa). The electrophoretic bands of PP remained largely unchanged after HIU alone, indicating that HIU did not alter the subunit composition of PP. However, under pH-shifting treatment alone or in combination with HIU, the bands corresponding to acid arachin intensified, and new bands appeared in the range of 15.5 to 18.0 kDa. suggesting that alkaline pH-shifting can lead to partial degradation of PP. Conarachin II and conarachin I are trimers, which can be dissociated into smaller subunits upon the addition of a reducing agent [50]. Thus, in the SDS-PAGE pattern of PP under reducing conditions, conarachin II dissociates into acid arachin (40.5, 37.5, and 35.5 kDa), as confirmed by Figure 3C.

Based on the SDS-PAGE analysis of the supernatant (Figure 3B), the conarachin I band intensity was notably darker after pH-shifting treatment, whether individual or combined. This demonstrates that the partial cleavage of peptide bonds is caused by pH-shifting rather than HIU, and the former can alter the structure of PP to a greater extent than the latter. Concurrently, samples treated with pH-shifting alone or in combination exhibited high molecular weight bands located at the upper part of the gel, with the intensity varying with HIU power. This indicates that some insoluble aggregates have been converted into soluble oligomers [51]. These may be connected by hydrogen bonds, as hydrophobic interactions and S-S bonds have been disrupted by SDS and β-mercaptoethanol (Figure 3D). Sun et al. [52] demonstrated that during the transformation of proteins into a molten globule state by pH-shifting, the cleavage of S-S bonds occurs, prompting the disassembly of protein aggregates into soluble oligomers.

### 3.5. Structural Characterization of PP

#### 3.5.1. Fourier Transform Infrared Spectroscopy (FTIR)

**Figure 4:**
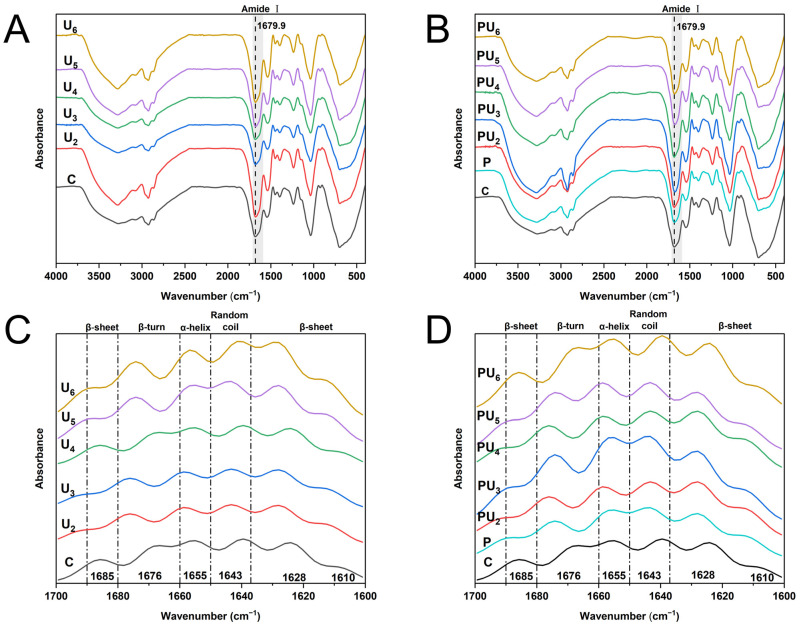
(**A**,**B**) Fourier transform infrared (FTIR) spectra and (**C**,**D**) deconvolution diagram of amide I band of native (C) and treated PP with high-intensity ultrasound (HIU, 200–600 W) and pH-shifting (pH 12), individually (U_2_, U_3_, U_4_, U_5_, U_6_, and P) or in combination (PU_2_, PU_3_, PU_4_, PU_5_, and PU_6_).

Building on our evidence suggesting that the structure of PP may have undergone alterations during the combined pH-shifting and HIU treatment, our immediate focus was on validating the impact of individual and combined treatments on the PP structure. FTIR spectroscopy is a reliable tool for analyzing molecular composition, structure, and intermolecular interactions, offering valuable insights into the secondary structure of proteins [53]. From Figure 4A,B, there were minimal changes in the FTIR spectra of PP after various treatments, suggesting the absence of notable alterations in the protein polypeptide backbone.

The amide I band (1700~1600 cm^−1^), corresponding to C=O stretching vibrations, exhibits sensitivity to alterations in the secondary structure of the protein [20]. Consequently, we performed deconvolution of the amide I band (Figure 4C,D) and quantified the relative abundance of secondary structures in PP following various treatments (Table 1). The results revealed varying extents of reduction in the α-helix and β-sheet contents, with an increase in β-turn and random coil structures. The combined treatments exhibited the most significant structural alterations (*p* < 0.05). Compared to native PP, the α-helix and β-sheet contents of combined-treated PP initially increased with increasing HIU power up to 300 W (PU_3_), while the β-turn and random coil contents decreased.

Generally, α-helices and β-turns constitute highly ordered and relatively stable structural elements [54]. A reduction in these structures could possibly be linked to the disruption of intrachain hydrogen bonds involving amino hydrogen and carbonyl oxygen (C=O) [49]. The β-sheet and random coil structures exhibit flexibility, with the β-sheet being relatively stretched and the random coil possessing non-repetitive and indeterminate characteristics [55]. Consequently, when the ultrasonic power is below 300 W, the PP structure transitions from a regular, rigid configuration to a disordered, flexible state as the power increases. These findings align with those presented by Kong et al. [56] for soy protein and Rafique et al. [57] for oat protein. The notable increase in β-sheet content and the accompanying decrease in β-turn and random coil structures observed when the HIU power surpassed 300 W may be related to the overprocessing effect caused by excessive HIU power, which induces protein refolding and even aggregation, thereby decreasing the proportion of flexible structures [58].

#### 3.5.2. Intrinsic Fluorescence Spectroscopy

**Figure 5:**
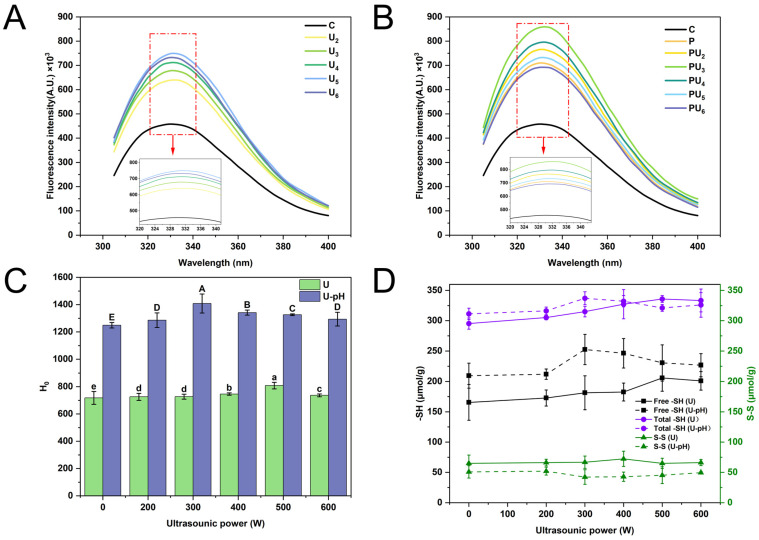
(**A**,**B**) intrinsic emission fluorescence spectra, (**C**) surface hydrophobicity (H_0_), and (**D**) sulfhydryl groups (-SH) and disulfide bonds (S-S) of native (C) and treated PP with high-intensity ultrasound (HIU, 200~600 W) and pH-shifting (pH 12), individually (U_2_, U_3_, U_4_, U_5_, U_6_, and P) or in combination (PU_2_, PU_3_, PU_4_, PU_5_, and PU_6_). U and U-pH represent HIU treatment alone and the combined treatment of HIU and pH-shifting, respectively. Different upper (A–E) and lower (a–e) case letters denote marked differences in the H_0_ of PP with HIU treatment alone and with a combination of pH-shifting and HIU, respectively (*p* < 0.05).

Intrinsic fluorescence spectroscopy is considered a robust indicator to evaluate changes in protein tertiary structure, indicating the extent of exposure of aromatic amino acids (Trp, Tyr, and Phe) to the polar environment [59]. The intrinsic fluorescence (Figure 5A,B) of PP increased with the increase in HIU power (*p* < 0.05). In comparison to individual HIU and pH-shifting treatments, the combined treatment demonstrated a more significant enhancement, reaching its maximum value in the PU_3_ treatment. Typically, the increased fluorescence intensity can be attributed to more extensive protein unfolding and the increased exposure of Tyr and Trp residues [60]. No red or blue shift was observed in any samples, indicating that the maximum emission wavelength remained unchanged.

However, the fluorescence intensity decreased after the U_6_ treatment, and the combined treatment showed a reduction at 400 W HIU power. This phenomenon may be attributed to overprocessing-induced overheating, leading to protein aggregation that obscures the exposed Tyr and Trp residues. Alternatively, excessive cavitation effects may quench the exposed fluorescent chromophores [61]. When protein is treated by pH-shifting during HIU it is more sensitive to the cavitation effects of HIU than either treatment alone, resulting in an earlier decline in fluorescence intensity [62].

#### 3.5.3. Surface Hydrophobicity (H_0_)

The H_0_ serves as an indicator of changes in the protein tertiary structure [63]. As depicted in Figure 5C, the H_0_ of PP initially increased markedly as the HIU power was increased (*p* < 0.05). The H_0_ of PU_3_ (14,083.00) was nearly twice as much as that of C (7181.43) (*p* < 0.05). Because the H_0_ is contingent upon the quantity of exposed hydrophobic groups on the protein surface that are accessible for binding [64], the increase in H_0_ is likely due to protein unfolding, exposing initially buried hydrophobic groups [65]. Higher H_0_ of PP correlates with increased solubility (Figure 1A), suggesting a reduction in interactions between hydrophobic patches on the protein surface, leading to a decrease in PP particle size. Similar conclusions were reached by Mozafarpour et al. [66] in their research. Furthermore, in line with the intrinsic fluorescence intensity, H_0_ decreased at excessive HIU power. This can be attributed to overheating or oxidation caused by excessive HIU, triggering protein denaturation and leading to the reburial of initially exposed hydrophobic groups.

#### 3.5.4. Sulfhydryl (-SH) and Disulfide Bonds (S-S)

The -SH and S-S contents provide further insight into protein structural changes. The contents of total -SH, free -SH, and S-S in PP are illustrated in Figure 5D. With increasing HIU power, a marked increase in the total -SH and free -SH contents was observed initially, accompanied by a decreasing trend in the S-S content, with combined treatments showing more pronounced alterations compared to individual treatments (*p* < 0.05). The initial increase in free -SH content can be attributed to two main reasons. The first is the cleavage of S-S bonds, leading to the formation of new -SH groups [67]. A concurrent increase in total -SH and a decrease in S-S also occur. The second is protein structural unfolding, leading to the exposure of internal -SH groups, which are then converted into free -SH groups [68]. Moreover, as shown in the SDS-PAGE results (Figure 3), conarachin II is a trimer formed by S-S bonds. It contains sulfur-containing amino acids, such as Cys and Met residues, whose sulfur atoms are highly reactive with oxygen-free radicals produced during oxidation [69,70]. This leads to the interchange reaction between -SH and S-S, which may also result in changes in the content of free -SH. In contrast, at relatively high HIU power (PU_4_), a reduction in free -SH content was observed. This may be attributed to the oxidation of increased -SH groups by radicals such as H• and •OH generated from cavitation effects, facilitating the formation of S-S bonds and thus rigidifying the previously loosened protein structure [33,71].

### 3.6. Emulsifying Properties

#### 3.6.1. Particle Size, Zeta Potential, and Storage Stability of PP Emulsion

**Figure 6:**
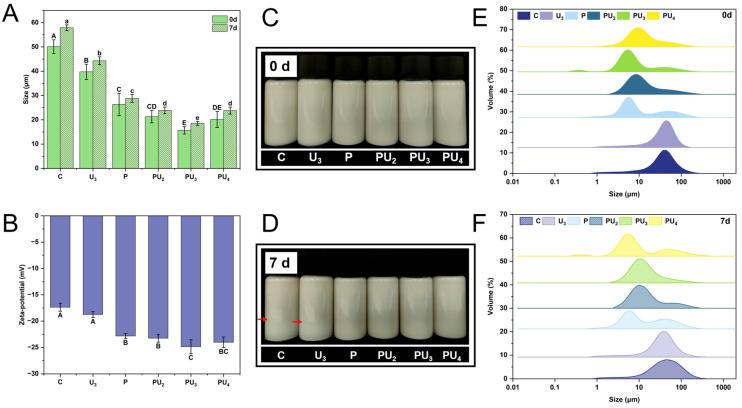
(**A**) Mean particle size, (**B**) zeta potential, (**C**,**D**) appearance, and (**E**,**F**) particle size distribution of fresh and 7-day-stored (**D**) emulsions stabilized by native (C) and treated PP with high-intensity ultrasound (HIU, 200–600 W) and pH-shifting (pH 12), individually (U_3_ and P) or in combination (PU_2_, PU_3_, and PU_4_). Different upper (A–E) and lower (a–e) case letters denote marked differences in the size and zeta potential of varying treatments, respectively (*p* < 0.05).

Proteins stabilize emulsions through two primary mechanisms: (1) adsorption at the oil–water interface to form a protective interfacial film, and (2) increasing the viscosity of the continuous phase, which retards droplet coalescence and phase separation [72,73]. The emulsifying property of proteins is closely related to their solubility, hydrophobicity, and conformational flexibility, as these factors govern the adsorption kinetics and interfacial film strength. In addition, the emulsifying property is also related to the protein concentration, which domains the viscosity and molecular interactions within the aqueous phase in emulsions [74,75]. Consequently, in light of the findings above, we chose C, U_3_, P, PU_2_, PU_3_, and PU_4_ for emulsion preparation, and we measured the particle size and droplet size distribution of emulsions in both fresh and 7-day stored conditions. From Figure 6B it can be noticed, although all fresh emulsions exhibit a uniform opalescent appearance, that the emulsion formulated with C has a larger particle size (50.09 μm) and a broader droplet size distribution (Figure 6A,E). Emulsions prepared from PP treated by HIU and pH-shifting individually (U_3_ and P) exhibited a significant reduction in particle size, with the droplet size distribution shifting to the left to varying degrees (*p* < 0.05). Notably, the combined treatment (PU_3_) resulted in the smallest particle size (15.68 μm, *p* < 0.05), demonstrating the synergistic effect of HIU and pH-shifting on enhancing the emulsifying properties of PP. This improvement can be attributed to the disruption of protein conformational structures by HIU and pH-shifting, which increases protein solubility and promotes protein adsorption at the oil–water interface. The reduced interfacial tension and the formation of a stable interfacial film contribute to the enhanced emulsion stability. Specifically, HIU and pH-shifting increase the surface hydrophobicity (H_0_) of proteins (Figure 5C), achieving a balanced hydrophobic-to-hydrophilic ratio essential for emulsification, which in turn promotes rapid adsorption of proteins at the oil–water interface. Additionally, the mechanical shear forces generated by HIU and the conformational changes induced by pH-shifting create a rigid and dense interfacial film, further stabilizing the emulsion [76,77].

After 7 days of storage, samples prepared from C and HIU-treated PP (individual treatment) demonstrated different levels of phase separation, with C showing a clearer lower liquid phase, and HIU-treated PP displaying a slight difference in clarity between the lower and upper layers. In contrast, the emulsions prepared from combined-treated PP maintained a uniform opalescent appearance (Figure 6D). Meanwhile, an increase in particle size led to a rightward shift or broadening of the droplet size distribution (Figure 6F). Nevertheless, the trend in particle size was consistent with that of the fresh emulsion, with PU_3_ remaining the smallest (18.53 μm, *p* < 0.05), indicating that the combined treatment of PP led to stabilized emulsions with superior storage stability (Figure 6A, 7d). The improved stability can be attributed to several factors. First, the reduction in protein aggregation and enhanced protein–water interactions increase the exposure of hydrophobic groups on the protein surface, augmenting steric hindrance and molecular flexibility. Smaller protein particles adsorb more readily at the oil–water interface, reducing interfacial tension and promoting better emulsification. Additionally, higher solubility accelerates the protein interfacial diffusion rate, further stabilizing the system [78,79].

The zeta potential, which reflects the overall surface charge of emulsion droplets, is a critical factor influencing emulsion stability. A higher absolute zeta potential value indicates stronger electrostatic repulsion between droplets, which prevents aggregation and enhances stability [80]. As shown in Figure 6B, emulsions prepared from PP subjected to individual HIU and pH-shifting treatments exhibit markedly greater absolute zeta potential values than the control emulsion (*p* < 0.05). The maximum value was observed in PU_3_ (−24.87 mV, *p* < 0.05), demonstrating the strongest electrostatic repulsion and the highest stability among all samples [81]. In PU_4_, however, the emulsion exhibits an enlarged particle size and a diminished absolute zeta potential, possibly owing to excessive exposure of hydrophobic groups, ultimately failing to form a continuous and uniform protein film around the droplets, leading to emulsion flocculation [82]. Similar observations were reported by Yu et al. [76], who found that overprocessing could result in a slight increase in emulsion droplet size due to protein reaggregation.

#### 3.6.2. PP Emulsion Microstructure Characterization

**Figure 7:**
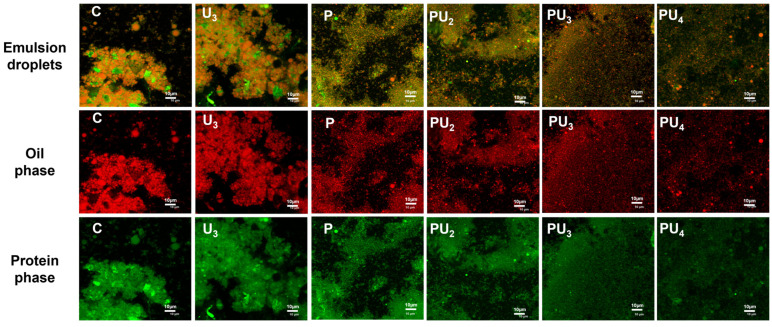
Confocal laser scanning microscopy (CLSM) micrographs of the emulsions stabilized by native (C) and treated PP with high-intensity ultrasound (HIU, 200–600 W) and pH-shifting (pH 12), individually (U_3_ and P) or in combination (PU_2_, PU_3_, and PU_4_).

CLSM was used to inspect the emulsion’s microstructure, thereby facilitating the observation of the distribution of oil droplets and proteins within the emulsion. From Figure 7, it can be observed that the oil droplets in the control emulsion are relatively larger and mostly exhibit an irregular spherical shape. This could be ascribed to the rigid structure of native protein aggregates, which possess poor surface characteristics, leading to slow diffusion rates and weak affinity for the oil–water interface [83]. Additionally, it can be observed from the images that although the control emulsion’s protein coats the oil droplets, the resulting interfacial film is discontinuous and non-uniform, with some droplets not being fully surrounded by the protein. Thus, we hypothesize that the instability of emulsions prepared with native PP is due to the variable size of oil droplets and the extent of protein aggregation, which together prevent the formation of a homogeneous and continuous oil-in-water structure. Similarly, Yang et al. [16] have proposed that the aggregation of EYP particles induces a non-uniform interfacial film on emulsion droplets, which in turn leads to a reduction in the film’s mechanical robustness and an increased likelihood of emulsion destabilization, characterized by enlarged droplet sizes. As observed in Figure 7, individual treatment with HIU or pH-shifting leads to a reduction in droplet size and a more homogeneous distribution. The combined treatment, PU_3_, which contrasts with the samples treated with HIU or pH-shifting individually, yields a diminished droplet size and fosters a more uniform dispersion. Concurrently, as evidenced by the protein phase image, the protein aggregates in PU_3_ are well-dispersed, allowing for homogeneous adsorption at the oil–water interface, which is more conducive to emulsion stability.

Figure 7 indicates that pH-shifting of PP appears to significantly enhance its emulsification property. This may be ascribed to the molten globule structure induced by pH-shifting. The increased hydrophobic surface area within the molten side chain structure leads to the formation of a stronger viscoelastic protein film at the oil–water interface. Proteins in the molten globule form lose tertiary interactions and fully packed amino acid side chains, exhibiting a disordered tertiary structure in a partially folded conformation, which can promote interactions among proteins, thereby augmenting the mechanical strength of the interfacial adsorption film [84,85]. Due to the increased flexibility of the protein structure, HIU treatment results in faster protein diffusion on the oil–water interface, as proteins with more exposed hydrophobic regions more readily interact with the oil phase. However, with increasing power (PU_4_), structural changes induced by HIU may hinder the interaction between exposed hydrophobic groups and oil droplets, adversely affecting emulsifying properties and hindering the development of a continuous and homogenous protein film surrounding the droplets, resulting in emulsion flocculation [82,86].

#### 3.6.3. PP Emulsion Rheological Properties

**Figure 8:**
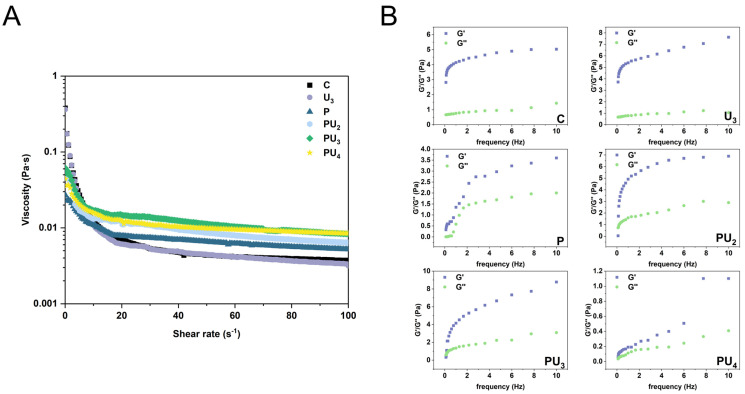
(**A**) Apparent viscosity and (**B**) frequency sweep of the emulsions stabilized by native (C) and treated PP with high-intensity ultrasound (HIU, 200–600 W) and pH-shifting (pH 12), individually (U_3_ and P) or in combination (PU_2_, PU_3_, and PU_4_).

Rheological properties are crucial indicators of emulsion performance and significantly influence emulsion stability. As shown in Figure 8A, the apparent viscosity of all samples sharply decreases as the shear rate increases, indicating shear-thinning behavior. This result may be attributed to the gradual stretching, deformation, or disintegration and rearrangement of emulsion structures under shear stress [87]. Alternatively, an increase in shear rate may lead to flocculation within the emulsion system, reducing the dispersive resistance between droplets, which in turn leads to a decrease in viscosity [88]. Emulsions prepared from PP treated with pH-shifting alone (P) exhibit higher apparent viscosity compared to those treated with HIU alone (U_3_) due to the stronger effect of pH-shifting on PP, which exposes more hydrophobic groups (Figure 5C), thereby improving interfacial properties. The emulsion prepared from combined-treated PP (PU_3_) exhibited the highest apparent viscosity among all samples. This is attributed to its smallest droplet size and most uniform distribution (Figure 7), which reduce droplet collision frequency and enhance emulsion stability, as supported by classical colloidal theory [89,90]. In accordance with Stokes’ law, an increase in the viscosity of the emulsion leads to a decrease in the droplet settling or floating rate, thereby enhancing the stability of the emulsion [91].

Figure 8B illustrates the frequency scan results for each emulsion. Both G′ and G″ of the emulsions show an increasing trend, indicating that PP treated with HIU and pH-shifting favors the development of a stable, viscoelastic film at the oil–water interface [26]. Within the linear viscoelastic region, G′ is greater than G″, indicating that the emulsion is more inclined to exhibit elastic behavior. With the combined treatment, G′ and G″ first increase and then decrease with increasing HIU power, reaching a maximum at 300 W (PU_3_). This is because pH-shifting loosens the PP structure, making it more sensitive to subsequent HIU treatment. By exposing more reactive groups and improving interactions between aggregated proteins, a thicker and denser interfacial film is formed, enhancing the viscoelasticity of the emulsion [92]. At 400 W, the excessive HIU treatment promotes protein aggregation, which weakens the strength and density of the protein network structure, thereby reducing the stability of the emulsion and leading to a decrease in viscosity, G′, and G″ [33].

## 4. Conclusions

The study substantiated that the combination of HIU and pH-shifting treatment exerts a synergistic effect on augmenting the solubility and emulsification properties of PP. Optimal modification parameters were identified as a pH_12_-shifting for 2 h in conjunction with 300 W power HIU treatment for 5 min. The solubility of PP increased significantly from 9.95% to 54.37%, while the emulsifying properties were markedly enhanced, as evidenced by the reduction in emulsion particle size to 15.68 μm in PU_3_. These improvements are attributed to the disruption of the rigid spherical structure of PP by pH-shifting, followed by the mechanical shear forces of HIU, which further unfold the protein and expose reactive groups, thereby enhancing solubility and interfacial adsorption. The combined treatment enables PP to adsorb more readily at the oil–water interface, generating a more robust viscoelastic interfacial film during emulsification. Future studies should explore how modified peanut proteins perform in formulated products like plant-based milk or dressings to evaluate their sensory attributes and functionality in real food matrices. The combination of pH-shifting and HIU not only improves the utilization of PP as a byproduct of peanut oil extraction but also broadens its application in the food field, contributing to resource sustainability.

## Figures and Tables

**Table 1:** Secondary structure contents of PP after high-intensity ultrasound, pH-shifting, and combined treatments.

	α-Helices (%)		β-Sheets (%)		β-Turns (%)		Random Coils (%)	
HIU (W)	U	U-pH	U	U-pH	U	U-pH	U	U-pH
0 (C)	24.43 ± 0.07 ^a^	21.09 ± 0.09 ^A^	42.54 ± 0.12 ^e^	43.22 ± 0.09 ^E^	15.25 ± 0.06 ^a^	13.70 ± 0.15 ^B^	17.79 ± 0.12 ^e^	21.99 ± 0.12 ^E^
200	22.66 ± 0.07 ^b^	19.97 ± 0.12 ^B^	42.73 ± 0.14 ^d^	44.27 ± 0.05 ^C^	14.88 ± 0.04 ^b^	13.08 ± 0.08 ^D^	19.73 ± 0.09 ^d^	22.68 ± 0.16 ^D^
300	22.31 ± 0.13 ^c^	18.17 ± 0.08 ^E^	42.84 ± 0.12 ^a^	45.64 ± 0.03 ^A^	14.79 ± 0.13 ^b^	12.24 ± 0.05 ^F^	20.06 ± 0.06 ^c^	23.95 ± 0.11 ^A^
400	21.82 ± 0.07 ^d^	18.91 ± 0.10 ^D^	42.96 ± 0.05 ^c^	44.82 ± 0.09 ^D^	13.83 ± 0.08 ^c^	12.90 ± 0.04 ^E^	21.39 ± 0.05 ^b^	23.37 ± 0.11 ^B^
500	21.75 ± 0.02 ^d^	19.71 ± 0.13 ^C^	43.41 ± 0.05 ^b^	43.92 ± 0.09 ^B^	13.48 ± 0.11 ^d^	13.22 ± 0.11 ^C^	21.36 ± 0.06 ^b^	23.15 ± 0.07 ^C^
600	20.77 ± 0.03 ^e^	19.60 ± 0.06 ^C^	43.97 ± 0.14 ^a^	43.18 ± 0.16 ^E^	13.43 ± 0.15 ^d^	14.05 ± 0.06 ^A^	21.83 ± 0.06 ^a^	23.18 ± 0.17 ^BC^

Data are presented as the mean ± standard deviation (SD) of triplicate experiments. U and U-pH represent HIU treatment alone and the combined treatment of HIU and pH-shifting, respectively. Different uppercase (A–E) and lowercase (a–e) letters indicate markedly differences in the secondary structure contents of PP with HIU treatment alone and with a combination of pH-shifting and HIU, respectively (*p* < 0.05).

## Data Availability

Data are contained within the article.

## Abbreviations

The following abbreviations are used in this manuscript:

PP	Peanut protein
HIU	High-intensity ultrasound
CLSM	Confocal laser scanning electron microscopy
SDS-PAGE	Sodium dodecyl sulfate-polyacrylamide gel electrophoresis
FTIR	Fourier transform infrared
H0	Surface hydrophobicity
-SH	Sulfhydryl groups
S-S	Disulfide bonds
